# Highly efficient removal of thallium(I) by facilely fabricated amorphous titanium dioxide from water and wastewater

**DOI:** 10.1038/s41598-021-03985-3

**Published:** 2022-01-07

**Authors:** Gaosheng Zhang, Jinglin Luo, Hanlin Cao, Shengping Hu, Huosheng Li, Zhijing Wu, Yuan Xie, Xiangping Li

**Affiliations:** 1 Key Laboratory for Water Quality and Conservation of the Pearl River Delta, School of Environmental Science and Engineering, Ministry of Education, Guangzhou University, Guangzhou, 510006 China; 2 School of Chemistry and Chemical Engineering, Guangzhou University, Guangzhou, 510006 China; 3 Guangzhou Huake Environmental Protection Engineering Co., Ltd., Guangzhou, 510655 China; 4 Technical Centre for Soil, Agriculture and Rural Ecology and Environment, Ministry of Ecology and Environment, Beijing, 100012 China; 5 Guangdong Provincial Key Laboratory of Radioactive and Rare Resource Utilization, Shaoguan, 512026 China

**Keywords:** Environmental monitoring, Pollution remediation

## Abstract

In this study, amorphous hydrous titanium dioxide was synthesized by a facile precipitation method at room temperature, aiming to effectively remove thallium(I) from water. The titanium dioxide prepared using ammonia as precipitant (TiO_2_^I^) is more effective for thallium(I) uptake than the one synthesized with sodium hydroxide (TiO_2_^II^). The TiO_2_ obtained particles are amorphous, aggregates of many nanoparticles and irregular in shape. The thallium(I) uptake increases with the rise of solution pH value. Under neutral pH conditions, the maximal thallium(I) adsorption capacities of TiO_2_^I^ and TiO_2_^II^ are 302.6 and 230.3 mg/g, respectively, outperforming most of the reported adsorbents. The amorphous TiO_2_ has high selectivity towards thallium(I) in the presence of multiple cations such as K^+^, Ca^2+^, Mg^2+^, Zn^2+^ and Ni^2+^. Moreover, the TiO_2_^I^ is efficient in removing thallium(I) from real river water and mining wastewater. Additionally, the spent TiO_2_^I^ can be regenerated using hydrochloric acid solution and reused. The Tl(I) adsorption is achieved via replacing the H^+^ in hydroxyl group on the surface of TiO_2_ and forming inner-sphere surface complexes. Owing to its high efficiency, facile synthesis and environmental friendliness, the TiO_2_^I^ has the potential to be used as an alternative adsorbent to remove Tl(I) from water.

## Introduction

As a non-essential heavy metal to living organisms, thallium (Tl) has attracted more and more attentions because of its high toxicity^1–4^. Thallium occurs at very low levels in the natural aquatic environment^1,2^. However, anthropogenic activities such as coal combustion, mining and processing of Tl-hosting minerals lead to the release of large amount of Tl into natural water bodies, which pose a great threat to aquatic biota and human health^2^. To abate the health risk associated with exposure to thallium through drinking, stringent criteria for Tl concentration in water/wastewater have been established in many countries. For instance, in the United States, the USEPA has set 2 and 140 μg/L as the maximum Tl level in drinking water and wastewater discharged, respectively; in China, the limit of Tl in drinking water has been lowered to 0.1 μg/L and more stringent standard of 2 μg/L has been adopted as the discharge standard for industrial wastewater in some provinces^3,4^.

In aquatic environment, thallium usually exists in two oxidation states: thallous (I) and thallic (III)^3^. Tl(I) is considered to be very mobile and thus difficult to remove, because it generally forms most stable compounds in natural waters^1,5^. Therefore, in the thallium treatment domain most of the researches concerned Tl(I) removal. A variety of techniques including adsorption^6,7^, oxidation/precipitation^8–10^, ion exchange^11,12^, solvent extraction^13,14^, etc., have been used to treat Tl-containing water and wastewater. In comparation with other methods, adsorption has recently gained more and more attentions, due to the advantages of high efficiency, affordable cost, simple operation and little toxic sludge generation^3,6^. Numerous adsorbents such as carbon materials^15,16^, mineral materials^17,18^, biomass materials^19^, Prussian blue and analogues^20–22^, manganese oxides^23–27^ and titanium-based materials^28–30^, have been employed to remove Tl(I) from water or wastewater.

Titanium dioxide has been extensively investigated and used to remove heavy metal contaminants such as Cs(I), Cu(II), Pb(II), Cd(II), Ni(II), As(III), As(V), Cr(III), Cr(VI), U(VI) and Th(IV) from water or wastewater^31–35^, owing to its nontoxicity, affordable cost, good chemical stability and high affinity for these ions. However, little work has been done on the removal of Tl(I) with titanium dioxide. For instance, Tl(I) adsorption on anatase TiO_2_ (Degussa, P25) was studied by Kajitvichyanukul et al. and the maximal adsorption capacity was found to be only 6.3 mg/g under neutral pH conditions^36^; Asadpour et al. investigated the Tl(I) adsorption on anatase TiO_2_ nanoparticles synthesized via ultrasound method and found that its maximal adsorption capacity was 25 mg/g at pH 9.0^37^; Zhang et al. evaluated the Tl(I) adsorption on commercial rutile nano-TiO_2_ and determined that the maximum adsorption capacity was 51.2 mg/g at pH 7.0 ± 0.3^38^. Evidently, these well-crystalline TiO_2_ nanoparticles have relatively low Tl(I) adsorption capacity and are not feasible for Tl(I) removal. Therefore, it is vital and challenging to synthesize titanium dioxide with high-efficiency adsorption of Tl(I). The amorphous TiO_2_ may be a feasible choice because it often possesses abundant active sites, which are responsible for Tl(I) adsorption. Recently, a poor crystalline TiO_2_ had been prepared by a simple precipitation method in our laboratory. The as-synthesized TiO_2_ demonstrated a high maximal Tl(I) adsorption capacity of 239 mg/g at pH 7.0 ± 0.1^29^, which was remarkably superior to the well-crystalline TiO_2_. In addition, it could be easily synthesized in large scale. This is very interesting and the amorphous TiO_2_ might be a potential sorbent for effective Tl(I) removal because of its high performance, good chemical stability, cost-effectiveness, facile synthesis and environmental friendliness. However, to our best knowledge, the influence of precipitant used to prepare amorphous TiO_2_ on Tl(I) adsorption has never been investigated. Additionally, adsorption behavior and mechanism of Tl(I) on the amorphous TiO_2_ have never been systemically studied.

Hence, in this study, two different precipitants (NH_3_·H_2_O and NaOH) were used to synthesize the amorphous TiO_2_ via a facile precipitation method at room temperature. The synthesized TiO_2_ was characterized with a variety of techniques. The adsorption behaviors such as kinetics, isotherm, solution pH effect and coexisting cation influence were studied in details. Additionally, removal of thallium(I) from mining wastewater and natural river water was also evaluated. Moreover, a possible removal mechanism of thallium(I) was proposed.

## Materials and methods

### Materials

All chemicals such as Ti(SO_4_)_2_, NH_3_·H_2_O (30%), NaOH, NaNO_3_, TlNO_3_ and nano-TiO_2_ (P25) were purchased from Sinopharm Chemical Reagent Co. Ltd (Shanghai, China) and were analytical grade and used without further purification. Tl(I) stock solution was prepared by dissolving TlNO_3_ in deionized water. Prior to use, the working solution was freshly prepared by diluting Tl(I) stock solution to specified concentration with deionized water.

### Preparation of titanium dioxide

Titanium dioxide was prepared by a simple chemical precipitation method at room temperature. Briefly, 7.2 g Ti(SO_4_)_2_ was dissolved in a 200 mL deionized water. Under vigorously stirring, 10% ammonia solution or 1 M NaOH solution was then dropwise added to the Ti(SO_4_)_2_ solution until the pH was raised to approximately 7.5. The white precipitates produced were washed for several times using deionized water, then filtrated and dried at 55 °C for 24 h. The obtained titanium dioxides were denoted as TiO_2_^I^ (using NH_3_·H_2_O as precipitant) and TiO_2_^II^ (using NaOH as precipitant), respectively. In addition, titanium dioxide was also prepared by forced hydrolysis of Ti(SO_4_)_2_ at 70 °C for 4 h, and the as-prepared sample was denoted as TiO_2_^III^.

### Characterization

X-ray diffraction (XRD) analysis was performed on a PW3040/60 diffractometer (Philips Co., the Netherlands). The morphology of the synthesized and commercial TiO_2_ was observed with a Sigma 500 field scanning electron microscope (FESEM) (Carl Zeiss, Germany) and transmission electron microscope (TEM) (JEM-1230, JEOL, Japan). X-ray photoelectron spectra (XPS) were collected on an AXIS Supra spectrometer (Shimadzu Co., Japan) with a monochromatic Al Ka X-ray source (1486.6 eV). The XPS results were collected in binding energy forms and fitted using a nonlinear least-squares curve-fitting program (XPSPEAK41 Software).

### Tl(I) adsorption experiments

Batch tests were performed to estimate Tl(I) removal by the synthesized and commercial TiO_2_. Briefly, 10 mg TiO_2_ was added into 100 mL polyethylene bottles, which contain 50 mL Tl(I) solution with different concentrations. The solution pH was adjusted with 0.1 M NaOH and/or HNO_3_. The bottles were then sealed and were shaken on an orbital oscillator at 180 rpm for 24 h. Afterwards, supernatant was collected and filtered through a 0.45 µm membrane. More detailed description of adsorption tests is shown in the Supplementary Material.

### Tl(I) removal from real surface water and wastewater

To estimate the practicability of the synthesized TiO_2_, Tl(I) removal from real wastewater and spiked surface water was studied by batch experiments. The surface water was collected from the Pearl River near to Guangzhou University, China and the mining wastewater was sampled from a mining area, Guizhou Province, China. The river water pH value was 7.56 and spiked Tl(I) concentration was 20 μg/L. More detailed parameters of water quality were listed in Table S1. The pH value of mining wastewater was 2.73 and Tl concentration was 4.9 μg/L. More detailed parameters of water quality were summarized in Table S2. For the spiked river water, defined amount of TiO_2_^I^ (10 or 20 or 40 mg) was added into a 2000-mL beaker containing 1000 mL spiked Pearl River water. Afterwards, the solution was agitated by a magnetic stirrer at a speed of 200 rpm. 5 mL water sample was taken from the beaker at predetermined times. The samples were then filtered using a filter with 0.45-μm membrane. The residual Tl concentration was measured by an inductively coupled plasma mass spectrometry (ICP-MS). For mining wastewater, the test procedure was similar to the spiked river water.

### Analytical methods

Before analysis, the aqueous samples collected were acidified with HNO_3_ solution, and stored in glass bottles. Tl(I) concentration was determined by inductively coupled plasma mass spectrometry (ICP-OES, Avio 200, Perkin Elmer Co. USA). Trace level Tl was determined using an inductively coupled plasma mass spectrometry machine (ICP–MS, NexION 300, Perkin Elmer Co. USA).

## Results and discussion

### Characterization of TiO_2_

**Figure 1:**
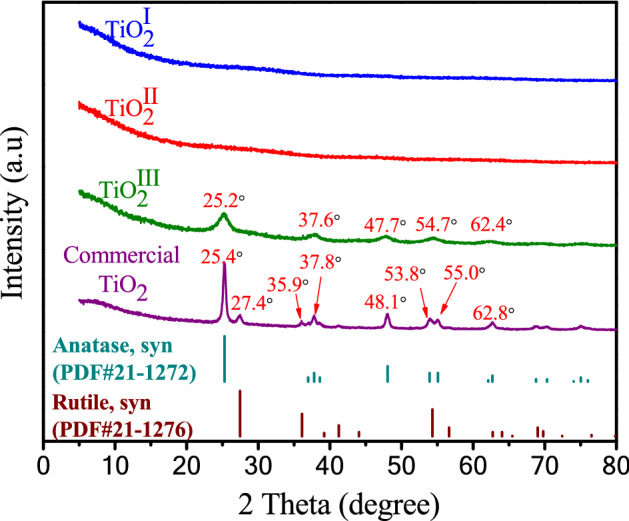
XRD patterns of synthesized and commercial TiO_2_.

**Figure 2:**
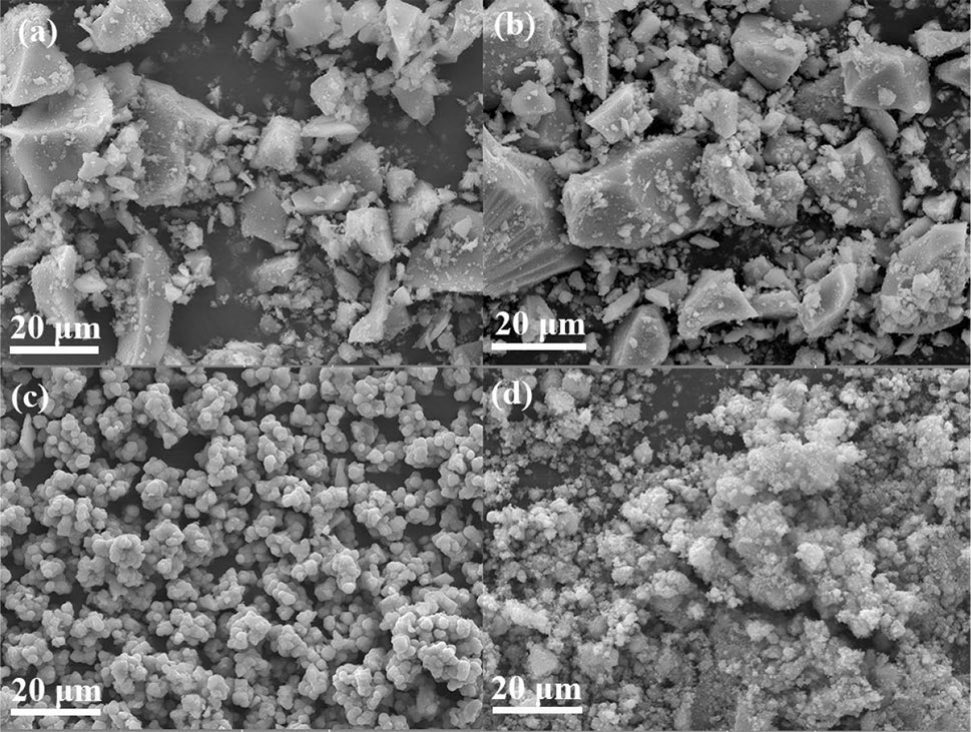
SEM images of TiO_2_^I^ (**a**), TiO_2_^II^ (**b**), TiO_2_^III^ (**c**) and commercial TiO_2_ (**d**).

**Figure 3:**
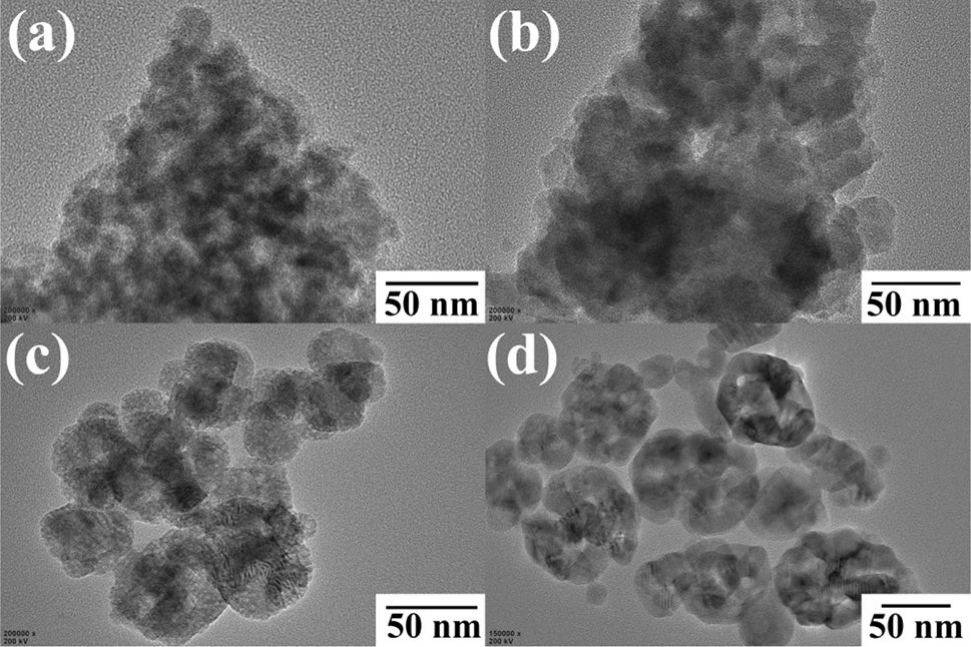
TEM images of TiO_2_^I^ (**a**), TiO_2_^II^ (**b**), TiO_2_^III^ (**c**) and commercial TiO_2_ (**d**).

Figure 1 shows the X-ray diffraction patterns of synthesized and commercial titanium dioxides. For TiO_2_^I^ and TiO_2_^II^, no obvious diffraction peaks can be observed, indicating that both of them are amorphous. Wang et al. had also synthesized amorphous TiO_2_ and observed similar phenomenon^39^_._ For TiO_2_^III^, five weak peaks appear at approximately 25.2, 37.6, 47.7, 54.7 and 62.4°, respectively, which are corresponding to the characteristic diffraction peaks of anatase (PDF#21-1272). This suggests that the anatase TiO_2_^III^ is not well-crystalline. For commercial TiO_2_, several strong peaks appear at 25.4°, 27.4°, 35.9°, 37.8°, 48.1°, 53.8°, 55.0° and 62.8°, respectively. The peaks at 25.4°, 37.8°, 48.1°, 53.8°, 55.0° and 62.8° coincide with those of anatase and peaks at 27.4 and 35.9° are in agreement with those of rutile, implying that the commercial TiO_2_ contains both well-crystalline anatase and rutile phase. Figure 2 exhibits the SEM images of the synthesized and commercial titanium dioxides. As can be seen, both TiO_2_^I^ and TiO_2_^II^ are irregular in shape and constituted by many small particles, while the TiO_2_^III^ demonstrates a regular sphere-like shape with a particle size of 1–3 μm. The commercial TiO_2_ particles are aggregates of smaller nanoparticles. TEM images of these four materials are demonstrated in Fig. 3. The TEM images of TiO_2_^I^ and TiO_2_^II^ further confirm that they are agglomerates of nanoparticles and amorphous. Relatively, the TiO_2_^III^ displays a polyhedron shape with certain crystallinity. The commercial TiO_2_ presents well-crystalline nanoparticles with particle size of about 15–30 nm.

### Adsorption isotherms

**Figure 4:**
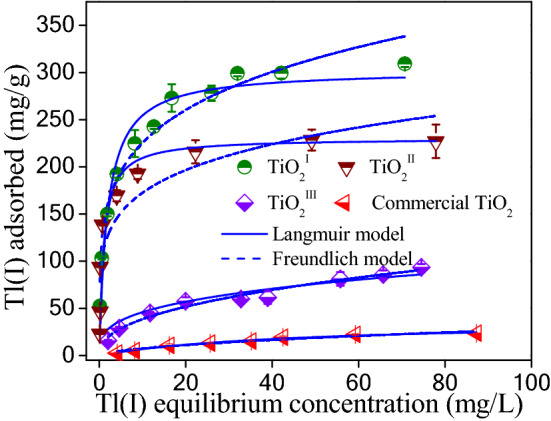
Adsorption isotherms of Tl(I) on the synthesized and commercial TiO_2_. Experiment conditions: Adsorbent dosage = 0.2 g/L; pH = 7.0 ± 0.1 and T = 25 ± 1 °C.

To evaluate the Tl(I) adsorption capacities of the synthesized and commercial titanium dioxides, the adsorption isotherm experiments were conducted at neutral circumstance. The results are illustrated in Fig. 4. Clearly, the adsorption capacities of synthesized titanium dioxides are far higher than that of the commercial one. Furthermore, the TiO_2_ synthesized via chemical precipitation has much higher adsorption capacity than the one prepared by forced hydrolysis. The differences in the maximum adsorption capacity between them might be ascribed to their crystallinity. The amorphous TiO_2_ may have more surface hydroxyl groups than well-crystalline TiO_2_, which are responsible for the Tl(I) adsorption. Interestingly, both TiO_2_^I^ and TiO_2_^II^ are rather efficient for Tl(I) removal, particularly for low concentration of Tl(I). In addition, it can be seen that the precipitant used to synthesize amorphous TiO_2_ has great effect on Tl(I) adsorption. The TiO_2_ obtained using NH_3_·H_2_O as precipitant is much more effective for Tl(I) adsorption. However, extensive use of NH_3_·H_2_O might lead to ammonia pollution. The experimental data were fitted by the Langmuir model (Eq. (S1)) and Freundlich model (Eq. (S2)). The fitting curves are demonstrated in Fig. 4 and the adsorption constants obtained from the isotherms are listed in Table 2. It can be observed that the Langmuir model is more suitable for describing the adsorption behavior, due to the higher regression coefficients (Table 1). This indicates that the Tl(I) adsorption on the TiO_2_ follows a monolayer adsorption process, since the Langmuir model assumes that adsorption is limited to one monolayer. The maximal adsorption capacities of TiO_2_^I^, TiO_2_^II^, TiO_2_^III^ and commercial TiO_2_ are 302.6, 230.3, 106.3 and 34.7 mg/g at pH 7.0, respectively. A comparison between the synthesized TiO_2_ and adsorbents reported in literature for Tl(I) adsorption has been done (Table 2). Evidently, both TiO_2_^I^ and TiO_2_^II^ are more competitive than the majority of reported adsorbents, implying that amorphous TiO_2_ is a promising alternative for Tl(I) removal from water. Therefore, investigation was focused on the TiO_2_^I^ and TiO_2_^II^ in the following sections.

**Table 1:** Langmuir and Freundlich isotherms parameters for Tl(I) adsorption on the synthesized and commercial titanium dioxides at pH 7.0 ± 0.1.

Adsorbent	Langmuir model			Freundlich model		
	*q*_max_ (mg g^−1^)	*k*_L_ (L mg^−1^)	*R*^2^	*k*_F_ (L g^−1^)	1/*n*	*R*^2^
TiO_2_^I^	302.6	0.528	0.951	139.7	0.208	0.949
TiO_2_^II^	230.3	0.989	0.896	111.6	0.188	0.845
TiO_2_^III^	104.3	0.059	0.933	22.4	0.312	0.921
Commercial TiO_2_	36.7	0.023	0.984	2.0	0.569	0.944

**Table 2:** Comparison of Tl(I) maximal sorption capacities for different adsorbents.

Adsorbent	Tl equil. con. range (mg/L)	Dosage (g/L)	pH	Max. sorption capacity (mg/g)	References
TiO_2_ (Degussa, P25)	0–160	2.0	7.0	6.3	^36^
TiO_2_ nanoparticles	0–30	0.67	9.0	25	^37^
Nano-TiO_2_	0–12	–	7.0	51.2	^38^
TNTs	0–55	0.2	5.0	709.2	^28^
TNM-30	0–550	0.25	8.0	710.4	^30^
Titanium peroxide	0–70	0.2	7.0	412	^29^
MnO_2_	0–102	0.5	5.0	349	^23^
FeOOH-loaded MnO_2_	0–140	0.4	7.0	450	^40^
Fe–Mn binary oxide	0–400	0.5	10.0	236.4	^41^
MnO_2_@pyrite cinder	0–40	0.5	12.0	320.1	^42^
Alginate-PB	0–400	1.0	4.0	103	^22^
MFBC	0–300	1.0	6.0	170	^7^
TFNPs	0–50	0.1	7.0	111.3	^43^
Biochar	0–300	2.0	6.5	178.4	^44^
ZnKFeCN@Fe_3_O_4_ composite	0–160	0.6	7.0	120	^45^
Commercial TiO_2_	0–80	0.2	7.0	36.7	Present study
TiO_2_^III^	0–80	0.2	7.0	104.3	Present study
TiO_2_^II^	0–80	0.2	7.0	230.3	Present study
TiO_2_^I^	0–80	0.2	7.0	302.6	Present study

### Tl(I) adsorption kinetics

**Figure 5:**
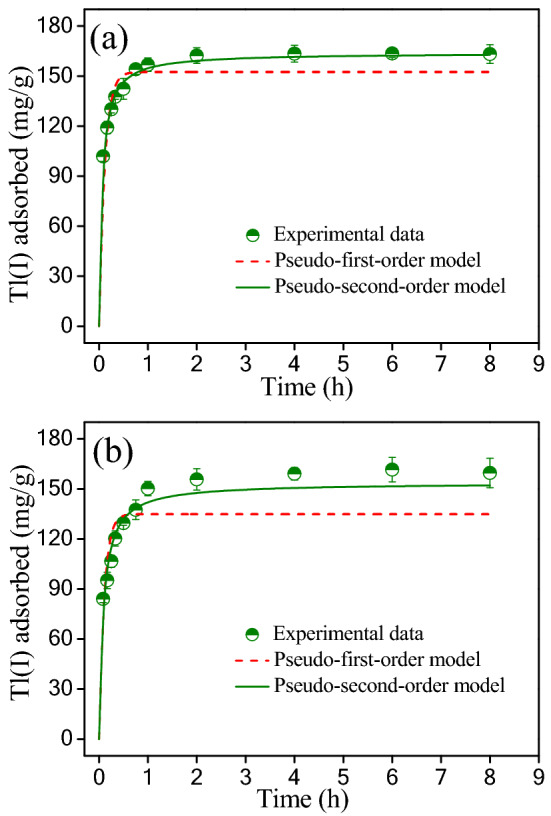
Kinetics of Tl(I) adsorption on TiO_2_^I^ (**a**) and TiO_2_^II^ (**b**). Experiment conditions: Initial Tl(I) concentration = 34.5 mg/L; adsorbent dosage = 0.2 g/L; pH = 7.0 ± 0.1 and T = 25 ± 1 °C.

Figure 5 presents the adsorption kinetics data of Tl(I) on the TiO_2_^I^ and TiO_2_^II^. A fast adsorption of Tl(I) was observed within the first 0.5 h. During this period, about 87.3 and 81.1% of equilibrium Tl(I) adsorption capacity was achieved for TiO_2_^I^ and TiO_2_^II^, respectively. Afterwards, the Tl(I) adsorption rate became slower and the equilibrium was established within about 4 h. Both the pseudo-first-order model (Eq. (S3)) and pseudo-second-order model (Eq. (S4)) were initially applied to stimulate the kinetic data. The fitting curves are depicted in Fig. 5 and constants obtained from these two models are provided in Table 3. In terms of *R*^2^, it has been found that the pseudo-second order model fits better the kinetic data than the pseudo-first order model, indicating that removal process of Tl(I) by the synthesized TiO_2_ involves chemisorption.

**Table 3:** Kinetic parameters for Tl(I) adsorption on the TiO_2_^I^ and TiO_2_^II^ fitted with the pseudo first order and pseudo second order models.

Adsorbent	Pseudo first order model			Pseudo second order model		
	*q*_e_ (mg g^−1^)	*k*_1_ (h^−1^)	*R*^2^	*q*_e_ (mg g^−1^)	*K*_2_ (g mg^−1^ h^−1^)	*R*^2^
TiO_2_^I^	152.5	8.93	0.910	163.9	0.102	0.995
TiO_2_^II^	134.8	9.93	0.991	153.6	0.079	0.998

Adsorption process is complicated and multi-step, involving bulk diffusion (adsorbate transport from the bulk solution to the outer surface of the liquid film), film diffusion (from the outer surface of the liquid film to the surface of the solid adsorbent), intraparticle diffusion (from the surface of the adsorbent to the interior pores), and adsorption on the surface actives of solid adsorbent^46,47^. The pseudo-second order model is therefore limited in accuracy because it considers adsorption as a single, one-step binding process^48^. Thus, intraparticle diffusion model (Eq. (S5)) was further used to describe the experimental data. The fitting results are shown in Fig. S1. For both TiO_2_^I^ and TiO_2_^II^, the plot of *q*_t_ vs *t*^1/2^ can be divided into two linear segments, indicating that Tl(I) adsorption contains multiple steps. The first linear section is corresponding to the fast adsorption stage, which is mainly controlled by film diffusion. The second linear section is a slow stage, which is governed by the diffusion of Tl(I) from the surface of adsorbent into the micropores.

### Influences of pH and ionic strength on Tl(I) adsorption

**Figure 6:**
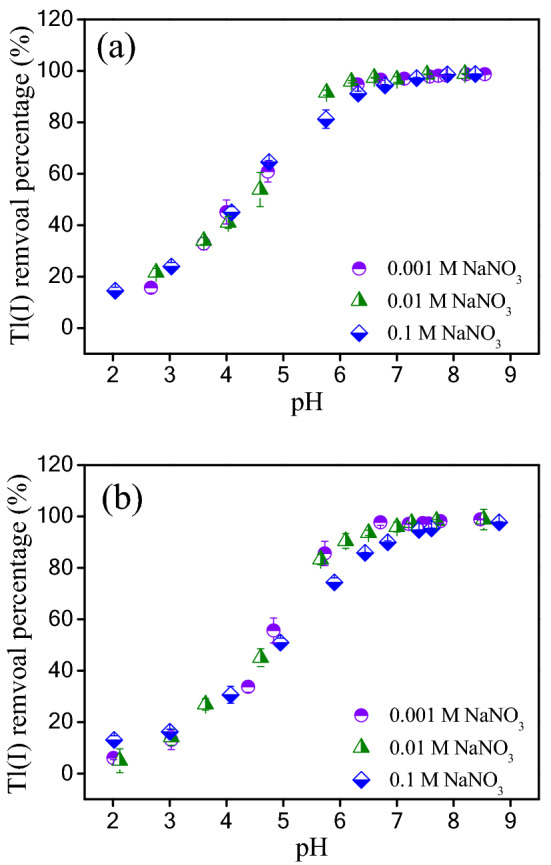
Effects of solution pH and ionic strength on Tl(I) adsorption by TiO_2_^I^ (**a**) and TiO_2_^II^ (**b**). Experimental conditions: Initial Tl(I) concentration: 30 mg/L; adsorbent dosage: 0.2 g/L; T: 25 ± 1 °C.

The solution pH affects not only the species of metal ions but also the surface functional group on the adsorbents for metal ions capturing^49^. Figure 6 demonstrates the influences of solution pH and ionic strength on Tl(I) adsorption. Evidently, Tl(I) adsorption on the TiO_2_^I^ and TiO_2_^II^ is strongly affected by the solution pH value, increasing gradually with its increase (2.0–9.0). And the optimal adsorption occurs under high alkaline circumstances. A similar trend was observed for the Tl(I) adsorption on titanium peroxide^29^ and titanium iron magnetic adsorbent^43^. In the tested pH range from 2.0 to 9.0, positively-charged Tl^+^ is the dominant species for Tl(I). Under acidic conditions, the surface of TiO_2_ was favorably protonated and consequently positively charged, which resulted in strong electrostatic repulsion between Tl^+^ and the positively-charged surface and depression of Tl^+^ sorption. With the increase in solution pH value, the TiO_2_ surface became less positively-charged and turned to be negatively-charged, which was beneficial for the sorption of Tl^+^. Thus, the uptake of Tl^+^ increased.

As can be seen in Fig. 6, the change in ionic strength (from 0.001 to 0.1 mM) did not greatly affect the adsorption of Tl(I) on both TiO_2_^I^ and TiO_2_^II^. Adsorption of ions by formation of outer-sphere complexes is very sensitive to the ionic strength change and always decreases with an increase in ionic strength, since the background electrolyte ions can also form this kind of complex via electrostatic force. On the contrary, adsorption by formation of inner-sphere complexes is insensitive to the variation of ionic strength^50^. Thus, it could be reasonably concluded that the Tl(I) was specifically adsorbed on the surface of TiO_2_ by formation of inner-sphere complexes.

### Influence of coexisting cations

Cations such as Ca^2+^, Mg^2+^ and K^+^ often exist in the surface water and groundwater^51,52^. Moreover, heavy metal ions such as Zn^2+^, Ni^2+^ and Cd^2+^ co-occur frequently with Tl^+^ in the mining and industrial wastewaters. These present cations might compete for the adsorptive sites on the surface of TiO_2_^I^ or TiO_2_^II^ with Tl^+^. Therefore, the influence of these cations on Tl^+^ adsorption was evaluated by batch tests at pH 4.5 ± 0.1.

**Figure 7:**
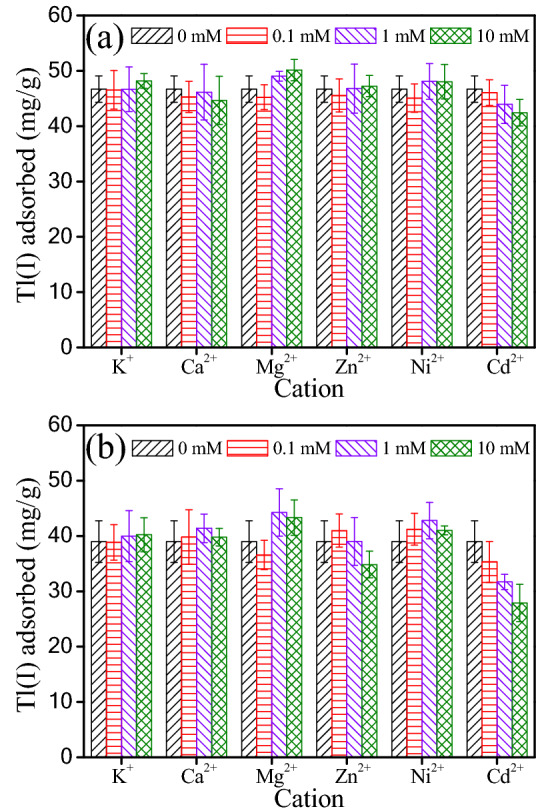
Influence of coexisting cations on Tl(I) adsorption by the TiO_2_^I^ (**a**) and TiO_2_^II^ (**b**). Experiment conditions: Tl(I) concentration = 18.5 mg/L; adsorbent dosage = 0.2 g/L; pH 4.5 ± 0.1 and T = 25 ± 1 °C.

Figure 7 shows the experimental results. Interestingly, the coexisting K^+^, Ca^2+^, Mg^2+^, Zn^2+^ and Ni^2+^ do not greatly affect the Tl^+^ adsorption and no great decrease is observed even though the concentration of present cations is as high as 10 mM. Awual et al. also found that the present K^+^ did not greatly prevent the adsorption of Cs^+^ (similar to Tl^+^ in chemical properties) by crown ether based conjugate material^53^. It is noteworthy that the coexisting Cd^2+^ inhibits Tl^+^ adsorption. For the TiO_2_^I^, this negative effect is slight and the Tl^+^ adsorption capacity still remains over 90% when the concentration of Cd^2+^ reaches up to 10 mM, being 100 times higher than that of initial Tl^+^. However, for the TiO_2_^II^, the negative influence is relatively remarkable and T(I) adsorption decreases by about 29% as the concentration of Cd^2+^ increases from 0 to 10 mM. Relatively, both TiO_2_^I^ and TiO_2_^II^ has high selectivity towards Tl(I) in the presence of multiple cations.

From above, we can see that the adsorption behaviors of Tl(I) on TiO_2_^I^ and TiO_2_^II^ are very similar. Therefore, we only studied the applicability, regeneration and mechanism of Tl(I) removal by the TiO_2_^I^ in the following sections.

### Tl(I) removal from real surface water and wastewater

**Figure 8:**
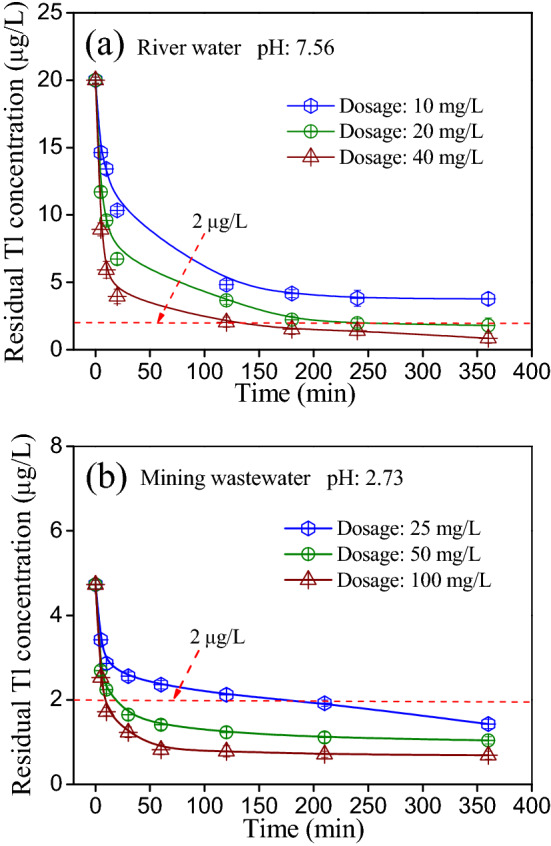
Kinetics of Tl(I) removal by TiO_2_^I^ from the (**a**) spiked Pearl River water and (**b**) mining wastewater.

To evaluate the applicability of the TiO_2_^I^, kinetics of Tl(I) removal from the surface water (spiked Pearl River water) and mining wastewater were respectively investigated by batch tests. The results are shown in Fig. 8a,b, respectively. For the Pearl River water, when the dosage of TiO_2_^I^ was 20 mg/L, the concentration of residual Tl(I) in the effluent was lowered to less 2 μg/L within 240 min (Fig. 8a). Tl(I) removal became more rapid as the dosage of TiO_2_^I^ increased. When the dosage was 40 mg/L, the residual Tl(I) reduced to less 2 μg/L within 120 min and below 1 μg/L at 360 min. For mining wastewater, when the dosage was 25 mg/L, the Tl concentration in the effluent was below 2 μg/L after treatment for 210 min. As the dosage increased to 50 mg/L, the residual Tl decreased rapidly from 4.7 to less 2 μg/L within 30 min (Fig. 8b). These results suggest that the TiO_2_^I^ is highly efficient for Tl(I) removal from the river water and real mining wastewater and has good applicability.

### Regeneration and reusability of TiO_2_^I^

**Figure 9:**
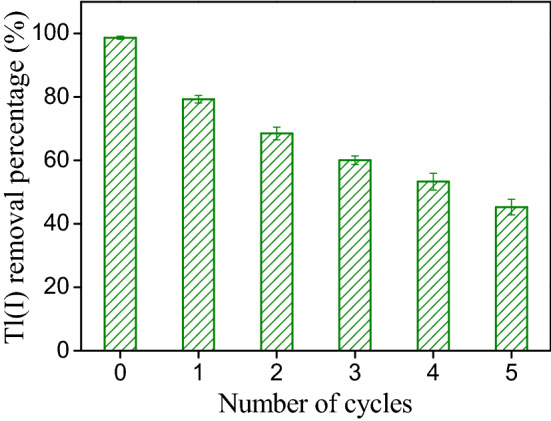
Variation of Tl(I) adsorption by the TiO_2_^I^ as a function of regeneration cycle.

The regeneration and reusability of adsorbent is an important factor affecting its use in real water treatment^54^. In order to assess the reusability of TiO_2_^I^, the Tl(I) desorption from spent adsorbent was investigated using 0.1 M HCl solution as desorbing agent and then the regenerated adsorbent was used in another adsorption–desorption cycle. Figure 9 illustrates the results of five consecutive adsorption/regeneration cycles. The cycle 0 is corresponding to the Tl(I) adsorption by the fresh TiO_2_^I^. As can be seen, the adsorption percentage of Tl(I) decreases with an increase in the number of cycles. After the first regeneration, the adsorption percentage of Tl(I) by the regenerated adsorbent reduces from 98.7 to 79.3%. This value is further lowered to 60.1% after the third regeneration and 45.3% after the fifth regeneration. Apparently, the reusability of TiO_2_^I^ is moderate, which may be ascribed to the relatively strong affinity between Tl(I) and the TiO_2_^I^. These results suggest that the TiO_2_^I^ could be regenerated but the times of reuse are limited.

### XPS analysis before and after Tl(I) adsorption

**Figure 10:**
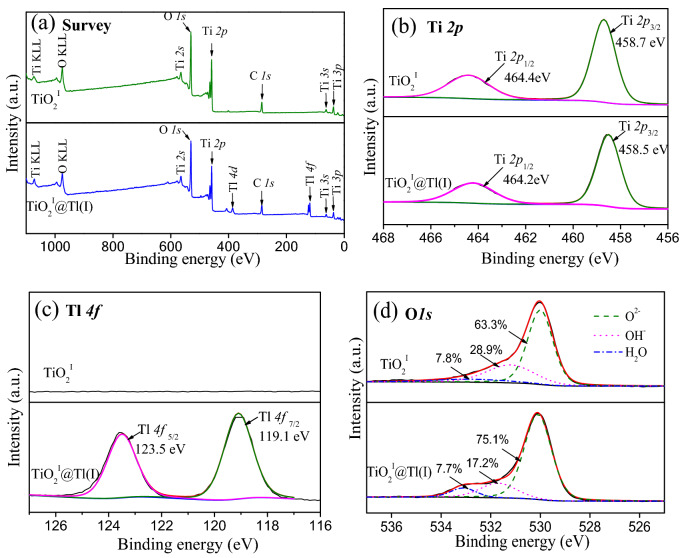
XPS spectra of TiO_2_^I^ before and after Tl(I) adsorption. (**a**) Survey spectra, (**b**) high-resolution Ti *2P* spectra, (**c**) high-resolution Tl *4f* spectra, (**d**) high-resolution O *1s* spectra.

In order to reveal the mechanism of Tl(I) adsorption by the TiO_2_^I^, XPS spectra of the TiO_2_^I^ before and after Tl(I) uptake were determined and analyzed. Figure 10a presents the survey spectra of the original and Tl(I)-sorbed TiO_2_^I^. Characteristic Ti peaks including Ti *2p*, Ti *2s*, Ti *3p*, Ti *3s* and Ti KLL along with O peaks are observed in the spectra of the original TiO_2_^I^. After reaction with Tl(I), two characteristic Tl peaks of Tl *4f* and Tl *4d* appear, suggesting that Tl(I) was adsorbed on the surface of TiO_2_^I^. High resolution XPS spectra of Ti *2p*, Tl *4f* and O *1s* of the pristine and Tl(I)-loaded TiO_2_^I^ are illustrated in Fig. 10b–d, respectively. The doublet peaks of Ti *2p*_3/2_ and Ti *2p*_1/2_ are located at 458.7 eV and 464.4 eV, respectively, indicating that the oxidation state of Ti in the TiO_2_^I^ is + 4^29,55,56^. These two peaks exhibit a slight shift (0.2 eV) to lower binding energy after Tl(I) sorption, which might be ascribed to the presence of strong interaction between TiO_2_^I^ and Tl(I). The two peaks of Tl *4f*_7/2_ and Tl *4f*_5/2_ are located at 119.1 eV and 123.5 eV, respectively, indicating that the oxidation state of Tl sorbed is + 1^23^. Obviously, no Tl(I) oxidation occurs during its adsorption by TiO_2_^I^. The O *1s* spectra can be divided into three peaks situated at 530.2, 531.7 and 533.1, corresponding to lattice oxygen (O^2−^), surface hydroxyl (–OH), and sorbed water (H_2_O), respectively. For the virgin TiO_2_^I^, the contents of O^2−^, –OH and H_2_O are 63.3, 28.9 and 7.8%, respectively. After Tl(I) sorption, the content of H_2_O showed no significant change, while the content of –OH species decreased obviously from 28.9 to 17.2% and correspondingly, the content of O^2−^ increased from 63.3 to 75.1%. Obviously, the H^+^ in –OH group was replaced by the Tl(I) species during its removal.

**Figure 11:**
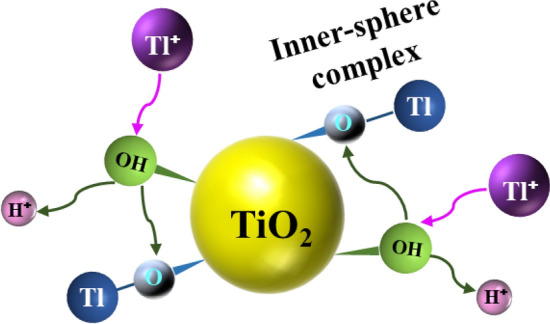
The proposed mechanism of Tl(I) adsorption on TiO_2_.

From the above-mentioned analysis, a possible mechanism of Tl(I) removal by the TiO_2_^I^ was established and the schematic diagram was illustrated in Fig. 11. Firstly, the Tl^+^ was transported to the surface of TiO_2_^I^ from bulk solution. Afterwards, the Tl^+^ replaced the H^+^ in –OH group on the surface of TiO_2_^I^ and an inner-sphere surface complex (Ti–O–Tl) was formed. Meanwhile, the H^+^ was released and entered into the bulk solution.

## Conclusions

Hydrous titanium dioxide was facilely synthesized by precipitation method and forced hydrolysis method, respectively. The TiO_2_ prepared at room temperature is amorphous and effective for Tl(I) adsorption, exhibiting high maximal adsorption capacities of 230.3–302.6 mg/g under neutral pH conditions. These values outperform the majority of reported adsorbents. The Tl(I) adsorption is strongly pH-dependent, increasing with an increase in solution pH value. The TiO_2_ has high selectivity for T(I) adsorption and it can be used repeatedly, though the times of reuse are limited. The mechanism of Tl(I) removal is that the H^+^ in –OH on the surface of TiO_2_ was replaced by Tl^+^ and inner-sphere surface complex was formed. The synthesized TiO_2_ has the potential to be used as an alternative adsorbent to remove Tl(I) from water, owing to its high efficiency, high stability, affordable cost, facile synthesis and environmental friendliness.

## Supplementary Information

Supplementary Information.
