# Supplementary material for: Highly efficient removal of thallium(I) by facilely fabricated amorphous titanium dioxide from water and wastewater
Source: Sci Rep. 2022 Jan 7;12:72. doi: 10.1038/s41598-021-03985-3 (PMC8741997; doi:10.1038/s41598-021-03985-3)
Supplement: Supplementary file 1 — Supplementary Information. [file 41598_2021_3985_MOESM1_ESM.docx]

**Supplementary Material**

**Highly efficient** **removal of thallium(I) by facilely fabricated amorphous titanium dioxide from water and wastewater**

Gaosheng Zhang ^1,*^, Jinglin Luo^1,3^, Hanlin Cao^4^, Shengping Hu^1^, Huosheng Li^1^, Zhijing Wu^1^, Yuan Xie^5^, Xiangping Li^2,*^

^1^School of Environmental Science and Engineering; Key Laboratory for Water Quality and Conservation of the Pearl River Delta, Ministry of Education, Guangzhou University, Guangzhou 510006, China (*Corresponding author, E-mail address: gszhang@gzhu.edu.cn)

^2^School of Chemistry and Chemical Engineering, Guangzhou University, Guangzhou 510006, China ( *Corresponding author, E-mail address: lxping@gzhu.edu.cn)

^3^Guangzhou Huake Environmental Protection Engineering Co.,Ltd., Guangzhou 510655, China

^4^Technical Centre for Soil, Agriculture and Rural Ecology and Environment, Ministry of Ecology and Environment, Beijing 100012, China

^5^Guangdong Provincial Key Laboratory of Radioactive and Rare Resource Utilization, Shaoguan, 512026, China

**Tl(I) adsorption** **experiments**

Tl(I) adsorption isotherm experiments were carried out using batch tests at pH 7.0 ± 0.1. Initial Tl(I) concentration varied from 5 mg/L to 130 mg/L. In each test, 10 mg TiO_2_ was loaded in a 100-mL polyethylene bottle containing 50 ml Tl(I) solution. The bottles were shaken on an orbit shaker at 170 rpm for 24 hours at 25 ± 1˚C. During the sorption process, the solution pH was adjusted several times with 0.1 M NaOH and/or HNO_3_ solution, to maintain at 7.0 ± 0.1. After adsorption, all water samples were filtered by a 0.45 µm membrane filter and analyzed for Tl(I).

Tl(I) adsorption kinetics experiments were carried out by batch tests. Defined amount of Tl(I) stock solution was added in a 2000-ml beaker and then a certain amount of deionized water was added, to make a 1000-ml Tl(I) solution of 34.5 mg/L. The solution pH was adjusted to 7.0 ± 0.1 by adding 0.1 M HCl and/or NaOH solution. Then, 0.2 g TiO_2_^I^ or TiO_2_^II^ was added to obtain a 0.2 g/L suspension. The mixture was stirred with a magnetic stirrer, and the pH was maintained at 7.0 ± 0.1 throughout the experiment by addition of dilute acid or base solution. Small aliquots (c.a. 5 ml) were taken from the media at certain time intervals. The samples were filtered through a 0.45 μm membrane filter before Tl analysis.

To examine the influence of pH and ionic strength on the Tl(I) adsorption, experiments were done over a wide range of pH 2.0–9.0. Initial Tl(I) concentration was 30 mg/L and the dosage of TiO_2_^I^ or TiO_2_^II^ was 200 mg/L. The ionic strength of the solutions varied from 0.001 M to 0.1 M by adding NaNO_3_. The solution pH was adjusted every four hours with dilute HNO_3_ or/and NaOH solution to designated value during shaking process. After 24 h, the final solution pH was measured and the supernatant was filtered through a 0.45 μm membrane for Tl analysis.

The influence of coexisting cations such as Ca^2+^, Mg^2+^, K^+^, Zn^2+^, Ni^2+^ and Cd^2+^ on the Tl(I) adsorption was examined at pH 4.5 ± 0.1 by adding a certain amount of Ca(NO_3_)_2_, Mg(NO_3_)_2_, KNO_3_, Zn(NO_3_)_2_, Ni(NO_3_)_2_ and Cd(NO_3_)_2_ to Tl(I) solution, respectively. The cation concentration ranged from 0.1 to 10 mmol/L. 10 mg TiO_2_^I^ or TiO_2_^II^ was added into a 100-ml polyethylene bottle, containing 50 ml of 18.5 mg/L Tl(I) solution. After agitation at 170 rpm for 24 h, the supernatant was filtered by a 0.45 μm membrane prior to Tl analysis.

**Langmuir model and Freundlich model**

$q_{\text{e}}=\frac{q_{\max}K_{\text{L}}C_{\text{e}}}{1+K_{\text{L}}C_{\text{e}}}$ (S1)

$q_{\text{e}}=K_{\text{F}}C_{\text{e}}^{1/n}$ (S2)

Where *q*_e_ and *q_max_* represent the amount of equilibrium adsorption capacity and the maximum adsorption capacity (mg·g^-1^), respectively; *C*_e_ is the equilibrium solution concentration (mg·L^-1^); *K*_L_ (L·mg^-1^) is the Langmuir coefficient; *K*_F_ is roughly an indicator of the adsorption capacity; n is the heterogeneity factor which has a lower value for more homogeneous surfaces

**Pseudo-first-order model and pseudo-second-order model**

$q_{\text{t}}=q_{\text{e}}(1-\text{e}^{-k_{1}t})$ (S3)

$q_{\text{t}}=\frac{q_{\text{e}}^{2}k_{2}t}{1+q_{\text{e}}k_{2}t}$ (S4)

Where *q*_e_ and *q*_t_ are the adsorbed amounts of Tl(I) per unit weight of the sorbent (mg/g) at equilibrium and at time t (h), respectively, *k*_1_ (h^-1^) and *k*_2_ [mg/(g·h)] are the pseudo-first-order and pseudo-second-order rate constants, respectively.

**Intraparticle diffusion model**

$q_{\text{t}}=k_{\text{p}}t^{0.5}+c$ (S5)

where *q*_t_ is the adsorbed amount of Tl(I) per unit weight of the sorbent at any time t (h). *k*_p_ is the rate constant, *C* is a constant, representing the effect of boundary layer. The values of *k*_p_ and *C* could be obtained from the plot of *q*_t_ versus *t*^0.5^.

Fig. S1. Kinetics of Tl(I) adsorption on TiO_2_^I^ (a) and TiO_2_^II^ (b) fitted with the intraparticle diffusion model. Experiment conditions: Initial Tl(I) concentration = 34.5 mg/L; adsorbent dosage = 0.2 g/L; pH = 7.0 ± 0.1 and T = 25 ± 1 °C.

Table S1. Characteristics of the Tl(I) spiked Pearl River water.

| Items | Value |
| --- | --- |
| pH | 7.56 |
| Na^+^ (mg/L) | 2.47 |
| K^+^ (mg/L) | 1.12 |
| Ca^2+^ (mg/L) | 14.73 |
| Mg^2+^ (mg/L) | 2.19 |
| NO_3_^-^ (mg/L) | 1.31 |
| Cl^-^ (mg/L) | 6.55 |
| SO_4_^2-^ (mg/L) | 19.14 |
| F^-^ (mg/L) | 0.26 |
| Mn^2+^ (mg/L) | 0.06 |
| Tl(I) (µg/ L) | 20 |

Table S2. Characteristics of the mining wastewater.

| Items | Value |
| --- | --- |
| pH | 2.73 |
| Na (mg/L) | 74.5 |
| K (mg/L) | 3.8 |
| Ca (mg/L) | 433.5 |
| Mg (mg/L) | 465.7 |
| Mn (mg/L) | 250.2 |
| Ni (mg/L) | 1.4 |
| Fe (mg/L) | 51.9 |
| Al (mg/L) | 25.6 |
| NO_3_^-^ (mg/L) | 5.3 |
| SO_4_^2-^ (mg/L) | 10846 |
| Cl^-^ (mg/L) | 4.8 |
| Tl (µg/ L) | 4.73 |
